# Impact of tumor volume and systemic therapy on outcome in patients undergoing IMRT for large volume head neck cancer

**DOI:** 10.1186/1748-717X-6-120

**Published:** 2011-09-22

**Authors:** Gabriela Studer, Tamara Rordorf, Christoph Glanzmann

**Affiliations:** 1 Department of Radiation Oncology, University Hospital Zurich, Raemistrasse 100, 8091 Zurich, Switzerland; 2 Department of Oncology, University Hospital Zurich, Raemistrasse 100, 8091 Zurich, Switzerland

**Keywords:** loco-regionally advanced HNC, tumour volume as prognostic factor, systemic therapy in advanced HNC, IMRT in advanced HNC

## Abstract

**Background:**

Former prospective analyses revealed gross tumor volume (GTV) as the most reliable parameter to statistically significantly predict disease control in head neck cancer (HNC) patients treated with definitive intensity modulated radiation therapy (IMRT) +/-concomitant systemic therapy. The most 'unfavourable' subgroup was characterized by total GTV (tGTV) of > 70 cc, translating in ~50 and 65% 3-year disease free (DFS) and overall survival (OAS, vs 68% and 88% in tGTV < 70 cc, p = 0.001 and 0.0001), and ~25% distant spread (vs 6% for tGTV < 70 cc, p < 0.0001).

The aim of this report was to analyze whether there is a subgroup out of patients with tGTV > 70 cc, which only marginally benefits from intensive curative treatment.

**Results:**

Between 03/2002-03/2011, 112 HNC patients with tGTV > 70 cc were definitively irradiated with curative intention. Mean tGTV was 104 cc (71-251). 98/112 (88%) patients underwent systemic therapy. Parameters with potential impact on disease outcome were retrospectively tested. The 3-year local-regional control (LRC), DFS and OAS rates were 61%, 50%, and 58%. The used cut-off value of 130 cc revealed an inverse association between tGTV and outcome. Patients able to undergo any systemic therapy (n = 98, mean tGTV0 103 cc, mean age 60 years) showed a satisfying and significantly superior outcome compared to the subgroup with radiation alone (n = 14, mean tGTV 99 cc, mean age 73 years), with 53% vs 17% 3-year DFS (p = 0.01). Radiation alone for tGTV > 130 cc failed to aim its curative goal in 3/3 patients.

**Conclusion:**

Patients with tGTV > 70 cc unable to undergo any systemic therapy represented a subgroup in which disease control was achievable in < 20% following curatively intended IMRT. Prospective testing of a larger sample size is needed to evaluate, if radiation alone for tGTV >~130 cc fails to meet its curative aim.

## Background

Former analyses revealed gross tumor volume (GTV) as the most reliable prognostic parameter for outcome in head and neck cancer (HNC) patients treated with simultaneous integrated boost intensity modulated radiation therapy (SIB-IMRT) +/- concomitant systemic therapy [1]: the 'unfavourable' subgroup was characterized by total GTV (tGTV) of > 70 cc, translating in ~50 and 65% 3-year disease free (DFS) and overall survival (OAS, vs 68% and 88% for tGTV < 70 cc, p = 0.001/0.0001), with distant spread in ~25% (vs 6% if tGTV < 70 cc, p < 0.0001) [2].

This 'unfavourable' subgroup represents one quarter of all squamous cell carcinoma (SCC) HNC patients referred to our institution for definitively IMRT in curative intention (112/458 (23%), 03/2011). Many patients presenting with such advanced tumours are suffering form alcohol abuse with related co-morbidities and frequently limited compliance. For this cohort with considerable risk for treatment failure and tolerance problems, the risk-benefit ratio of intensive treatment approaches with curative intention remains difficult to estimate in advance. Our philosophy is to try to prevent patients from usually quickly developing severe loco-regional symptoms due to large tumor volumes.

The aim of this report was to analyze whether there is a subgroup identifiable which only marginally benefits from intensive curative treatment.

Between March 2002 and March 2011, 112 HNC patients with tGTV > 70 cc were treated using a prospectively defined curative radiation therapy schedule. Patients diagnosed with other than squamous cell carcinoma or with nasopharyngeal cancer were excluded (n = 24). In 98/112 patients (88%), systemic therapy was administered. Table 1 shows patient and tumour characteristic. A second (n = 24), third (n = 2) or even fourth (n = 1) malignancy was diagnosed prior or after completion of treatment of the HNC in 27 patients of the cohort (24%). Twelve patients (10%) were initially diagnosed with or suspicious for small distant metastases (lung in most cases). This was not considered a contra-indication for a curative loco-regional IMRT approach. Patients with small or questionable M1 lesions were included into this analysis, as for those patients the same difficult question (loco-regionally curative treatment or not?) has to be answered in the clinical routine, and as suspicion for/limited initial M+ status did not turn out as an inverse parameter allowing to exclude patients from loco-regionally curative treatment (see results).

**Table 1:** Characteristics of the analyzed cohort

Parameters	Patients
**N patients included**	112 (100%)
**definitive SIB-IMRT**	112 (100%)
**conc. syst Tx, IC, no syst Tx**	75 (70%), 24 (21%), 14 (12%)
**time interval**	3/2002 - 03/2011
**male/female (%)**	96/16 (86/14)
**age**, mean/median (range)	62/61 years (33-87)
**WHO **performance status 0/1/2	78 (70%)/33 (29%)/1 (1%)
**Diagnosis (%)**	
oropharynx	62 (55%)
hypopharynx	22 (20%)
oral cavity	10 (9%)
larynx	5 (4%)
others	9 (8%)
CUP	4 (4%)
**T **(number)**, % **recurrence	5, 4% (101 cc, 10-206)
(mean volume, range)	
CUP	4, 4% (0 cc)
T1	6, 5% (7 cc, 4-13)
T2	15, 13% (34 cc, 5-63)
T3	23, 20% (60 cc, 10-122)
T4	59, 53% (77 cc, 12-206)

**N **(number)**, % **recurrence	1, 1% (5 cc)
(mean volume, range)	
N0	10, 6% (0 cc)
N1a-2b	39, 13% (34 cc, 1-125)
N2c	37, 64% (30 cc, 1-119)
N3	25, 23% (83 cc, 28-160)

**Gross Tumor Volumes **(GTV)	
mean primary GTV (range)	62 cc (0-206)
mean nodal GTV (range)	41 cc (0-160)
mean total GTV (range)	104 cc (71-251)

**Follow up**, mean/median (range) months	
all patients	26/21 (3-91)
alive patients (n = 58)	30/24 (5-85)
dead patients (n = 39)	18/13 (4-60)

SIB-IMRT: simultaneously integrated boost intensity-modulated radiation therapy

Conc. CT: concomitant chemotherapy

IC: induction chemotherapy

CUP: carcinoma of unknown primary

The selection of patients considered as 'still potentially loco-regionally radio-curable' remains somewhat arbitrary and influenced by personal experience, however, was consistent over the analyzed time period, as all patients were evaluated by the same radiation oncologists (CG and GS). Main criteria for the decision to support a curative treatment approach were 1) the anatomic tumor extent, including the GTV relation to surrounding critical structures, with respect to the boost dose volume, 2) oral cavity tumors (as definitive radiation of oral cavity tumors resulting in low disease control [3]), and 3) taking patients' compliance and interest in undergoing a time consuming therapy with risk for tolerance problems into account. In patients who presented with very advanced loco-regional disease with doubtful curative radio-therapeutic options, induction chemotherapy (IC) was provided if possible (n = 24). The aim of the IC was to ease the decision to initiate a potentially curative versus palliative loco-regional treatment approach, based on the response to IC [4].

### - Volumetric staging

In a former evaluation based on 88 retrospectively and 84 prospectively analysed IMRT patients (treated between 01/2002-12/2004 and 01-11/2005; n = total 172), the volumetric staging system (VSS) was found to represent the most important predictor for local-regional outcome [1]. This VSS bases on two cut-offs (15 cc and 70 cc), resulting in three volumetric subgroups: primary GTV or total GTV (tGTV) of 1-15 cc (favourable) vs 16--70 cc (intermediate) vs > 70 cc (unfavourable). While the primary GTV was used to predict local control rates, tGTV was shown to best predict nodal control, distant metastasis free, disease free and overall survival, respectively. The volumetric criterion has since been applied prospectively on our definitive IMRT patients to estimate disease outcome.

In the current work, we focus on the above described unfavourable subgroup with tGTV > 70 cc. Additional volumetric sub-grouping (cut-off 130 cc) and the impact of systemic therapy were retrospectively tested, aiming to try to identify a palliative subgroup out of the 'unfavourable' cohort with tGTV > 70 cc (outcome data were first analyzed by retrospectively testing two cut-offs, 100 cc and 130 cc, resulting in nearly identical outcome results for patients with tGTV 71-100 cc and 101-130 cc, while patients with tGTV > 130 cc did worse; this finding lead to the retrospective use of one single cut-off value of 130 cc).

Volumetric three-dimensional measurements (cc) of contoured GTVs were calculated by the Varian Treatment Planning System (TPS, Eclipse^® ^V8.5, Varian Medical Systems, Palo Alto, CA) volume algorithm.

## Methods

### - IMRT planning

Patients were immobilized from head to shoulders with commercially available thermoplastic masks and an individually customized bite block. CT images (2 mm slice thickness) were acquired from the upper aspect of the orbita to the level of the carina. Contrast agent enhancement was used whenever possible.

The target volumes were drawn on each axial planning CT slice, based on diagnostic CT images, supplemented with fused diagnostic MRI and/or PET-CT scans. The GTV included the gross extent of the primary disease and involved lymph nodes. PTV1 (planning target volume) was defined by adding a 1 - 15 mm margin to the GTV, dependent on the proximity of the lesion to critical structures. PTV2 covered areas at high risk for potential microscopic disease. PTV3 included the clinically negative cervical lymphatic nodes down to the supra-clavicular fossa (elective PTV). Organs at risk were outlined in three dimensions with an estimated planning organ-at-risk volume (PRV) margin of 2-10 mm. We used an extended-field IMRT (EF-IMRT) technique, where the primary was treated in one phase along with the regional lymph nodes. Irradiation was delivered with five or seven coplanar beam angles by a 6-MV dynamic multi-leaf collimator (MLC) system (sliding-window technique; Varian Medical Systems, Palo Alto, CA, USA). In some patients, volumetric modulated arc therapy (VMAT) technique on a Truebeam^® ^Varian linear accelerator was applied. All patients signed an informed consent as approved by our local ethical board.

### - Prescription Dose

As previously described [5], SIB-IMRT was performed using the following schedules (five fractions/week each):

• **SIB^2.00^**: daily dose 2.00 Gy (PTV1)/1.70 Gy (PTV2)/1.54 Gy (PTV3); total dose: 68-70.00 Gy (n = 20);

• **SIB^2.11^**: daily dose 2.11 Gy (PTV1)/1.80 Gy (PTV2)/1.64 Gy (PTV3); total dose: 69.60 -71.7 Gy (n = 76);

• **SIB^2.20^**: daily dose 2.20 Gy (PTV1)/2.00 Gy (PTV2)/1.80 Gy (PTV3); total dose: 66.00 -72.6 Gy (n = 16).

The dose was normalized to the mean dose in PTV1. For intensity optimization 95% of the prescribed dose should encompass at least 95% of the PTV and 100% of the prescribed dose encompassed the GTV. No more than 20% of any PTV would receive > 110% of its prescribed dose, while no more than 1% of any PTV would receive < 93% of the desired dose.

If the normal tissue volume or the exposed mucosal tissue volume was felt to be too large to receive 70 Gy by using the above described standard PTV1 (GTV plus 1-1.5 cm), the dose to PTV1 was reduced to 66-68 Gy, with no change to the prescription dose delivered to the GTV ('GTV-PTV').

### - Chemotherapy

#### a) Cisplatin

Systemic standard concomitant cisplatin based therapy was given to eligible patients (n = 76, 68%); our preferred cisplatin regimen of 40 mg/m2 i. v. per radiation week was used in all cases.

#### b) Cetuximab

Since April 2006, cetuximab has been used for patients who were not eligible to undergo cisplatin chemotherapy. Most frequent reasons for initial cetuximab therapy or switch from standard cisplatin based chemotherapy to cetuximab were decreased hearing, tinnitus, or impaired renal function (n = 26, 23%). An intravenous loading dose of 400 mg/m^2 ^cetuximab was administered in the week prior to the commencement of radiation, followed by 250 mg/m2 per radiation week.

#### c) Induction Chemotherapy (IC)

In 24 (21%) compliant patients with no serious medical contra-indications for IC, who presented with very advanced loco-regional disease (mean 120 cc, range 73-177) with poor curability, IC was offered. The aim of IC was to ease the decision to initiate potentially curative radiation therapy, based on the response to IC. Most frequently used IC drug combination was taxotere/cisplatin/5-fluoro-uracil.

### Statistics

The influence of the tumour volume (tGTV) and systemic therapy was retrospectively tested. In addition, univariate analysis of the impact of TN stages, age, diagnosis, distant metastasis at initial diagnosis, and pre-therapeutic performance status were performed. Statistical calculations were performed using the statistics program implemented in StatView^® ^(version 4.5; SAS Institute, Cary, NC). Proportions were compared using the Chi-square test. Univariate analyses were performed with a Cox proportional hazards regression model in StatView^®^. Actuarial survival data were calculated using Kaplan-Meier curves and log-rank tests implemented in StatView^®^. Stratification of variables was done with help of log rank (Mantel-Cox) calculation.

P values < 0.05 were considered statistically significant.

## Results

### - Disease control

**Figure 1:**
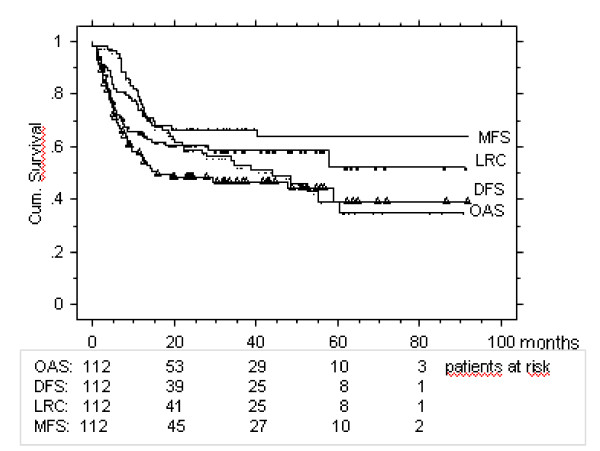
**Kaplan Meier actuarial survival rates of the entire cohort**. LRC: loco-regional control rate. MFS: metastasis free survival rate. DFS: disease free survival rate. OAS: overall survival.

After a mean/median follow up time of 26/21 months, 51 patients (46%) were alive with no evidence of disease (ANED) when last time seen. The related follow up times, time to treatment failure and Kaplan Meier actuarial survival curves are shown in Table 2, 3, and Figure 1.

**Table 2:** Duration of follow up and interval of disease free survival, analysed according to the status of patients when last time seen

Status	follow up, months	time to disease, months
last time seen (n)	mean/median (range)	mean/median (range)
**ANED **(51, 46%)	37/36 (3-91)	0
**INED **(10, 9%)	26/19 (5-60)	0
**AD **(12, 11%)	10/7 (2-23)	4/8 (0-11)
**DOD **(39, 35%)	16/12 (4-53)	5/4 (0-28)

ANED: alive with no evidence of disease

INED: inter-current dead without disease

AD: alive with disease

DOD: died of disease

na: not assessable

**Table 3:** Actuarial survival rates related to +/- systemic therapy and tGTV volume

Parameters (n)	3y-OAS	3y-DFS	3y-DMFS	3y-LRC
**entire cohort (112)**	58%	50%	70%	61%

**no systemic therapy (14)**	25%	17%	50%	25%
**any systemic therapy (98)**	61%	53%	72%	64%
*p-value*	*< 0.0001*	*0.01*	*0.1 (NS)*	*0.04*

**< 130 cc (91)**	58%	51%	75%	62%
**> 130 cc (21)**	30%	38%	40%	40%
*p-value*	*0.1 (NS)*	*0.2(NS)*	*0.001*	*0.05*

**no systemic therapy (14)**	25%	17%	50%	25%
**any systemic therapy, > 130 cc (19)**	30%	40%	50%	45%
**any systemic therapy, < 130 cc (79)**	63%	55%	78%	76%
*p-value*	*0.0001*	*0.03*	*0.01*	*0.04*

OAS: overall survival

DMFS: distant metastasis free survival

DFS: disease free survival

LRC: loco-regional control

Distant spread (M+) developed in 32/112 patients (29%); lesions suspicious for M+ were pre-therapeutically diagnosed in 12/32 patients (38%), which reached the same 2-year overall survival rate of 65% as the initially M0-subgroup; 8 of the 12 patients were still alive mean 11 months post treatment (4-21). In 15/32 (47%), distant metastases remained the only sign of disease (i.e., M+ with loco-regional control) mean 21 months (5-84) post IMRT. In 7 of 32 patients (6% of the entire cohort) new and isolated distant disease during the post-treatment follow up period were found.

Thirty-six patients (32%) experienced local failure, in 21 of them (58%), the primary tumour persisted (persistent disease defined as macroscopic persistence or re-growth to macroscopic disease during the first 3 months from treatment start). Nodal failure occurred in 25 (22%) patients, in 13/25 (52%) as persistent disease.

### - Impact of the tumour volume

The expected inverse association between tumour volume and disease control was tested using the retrospectively chosen cut-off value of 130 cc (Table 3): tGTV < 130 cc resulted in a tendency towards superior OAS, DFS and LRC rate, with a significant difference in the distant metastasis rate.

### - Impact of systemic therapy

**Figure 2:**
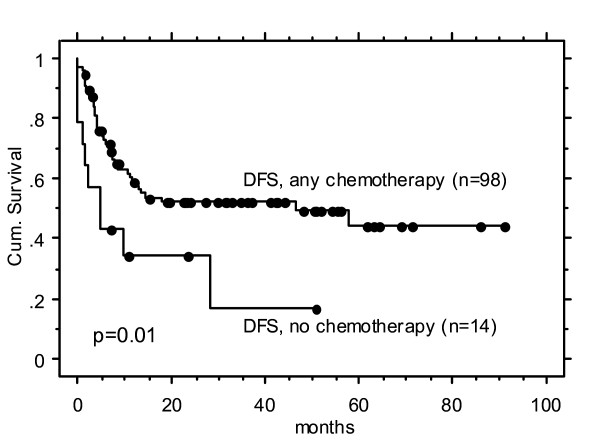
**Kaplan-Meier actuarial disease free survival (DFS) rates of subgroups with and without any systemic therapy**.

Patients who where eligible to undergo any systemic therapy (i. e., 1-7 cycles of concomitant cisplatin or cetuximab, and/or induction chemotherapy, n = 98 (88%)), versus those who were not (n = 14 (12%) - in most cases due to co-morbidity and/or age) were found to achieve significantly superior 3-year survival rates (Table 3, Figure 2**)**. The tGTV was similar in both groups (mean 99 cc vs 103 cc, NS), while the mean age expectedly significantly differed (60 years (range 41-81) in patients with combined modality treatment, vs 73 years (range 58-87) in the IMRT only subgroup, p = 0.01). Eleven of the 14 patients who underwent radiation only (79%), died from disease during the first 20 months; however, in 8 of them, substantial subjective loco-regional benefit was documented for a mean duration of 17 months (range 4-20). The remaining three patients who experienced no benefit suffered from persistent local disease. Three patients with tGTV > 130 cc treated with radiation alone died from disease after 2, 3, and 10 months, respectively.

Only 2/14 (14%) patients treated with IMRT alone remained alive with no evidence of disease, vs 41/78 (52%) treated with any systemic therapy for tGTV < 130 cc, vs 8/19 (42%) treated for tGTV > 130 cc, respectively.

### - Additional potentially prognostic parameters

The initial pre-treatment WHO performance status (Table 1) was not found to be of prognostic value (49% vs 50% 3-year DFS, p = 0.26). Similarly, there was no gender-related outcome difference (p = 0.3). Analysis based on T and N stages or TN grouping resulted in non-significantly different or nearly identical Kaplan Meier survival curves. Also the primary tumour site (mesopharynx versus hypopharynx) or age (grouped in decades: < 50 vs 50-60 vs 61-70 vs > 70 years) showed no prognostic value. Patients suspicious of distant metastasis did not worse compared with initially M0-patients (see above). Any potential impact of the human papilloma virus (HPV) status on the disease control in this 'very large tumour volume' patient cohort could not be assessed, as the HPV status was not available in most of our patients.

## Discussion

The previously defined 'unfavourable' SCC HNC patient subgroup with large tGTV > 70 cc [1] has been updated (Table 1, Figure 1) and further analyzed, using the advantage of volumetric staging to define further volumetric subgroups. The aim of this report was to analyze whether a subgroup of patients with large tGTV > 70 cc referred for curative treatment can be defined which only marginally benefits from intensive curative treatment. Weaknesses of this report are its retrospective approach, retrospective subgroup analysis, and the use of different chemotherapeutic schedules and chemotherapeutic dose-intensity, respectively. The strength of this report is a homogeneous IMRT contouring and treatment delivery performed according to prospectively defined treatment schedules in all patients, and in the relatively large sample size.

Our outcome results are comparable with selected recently published data on 'non-resectable' HNC treated with radio-chemotherapy, with DFS/OAS rates of approximately 50/60% versus ~25/45% [6], 65/70% [7], 50/65% [8].

We found a statistically significantly inferior outcome in patients not being able to undergo any systemic therapy (Table 3 and Figure 2). This result is limited by the unbalanced sample sizes (14 vs 98), and the significant difference in mean age (expectedly, as higher age and related frequent severe co-morbidity are usual reasons to not apply systemic therapy). It is known that higher age per se may inversely influence outcome [9] - age could therefore be an independent reason for inferior outcome. A potential difference of the impact of systemic therapy on very advanced ('unresectable') versus less advanced stages (lower tumor load) HNC is not well known to our best knowledge. The subgroup with tGTV > 70 cc and unable to undergo systemic therapy still achieved 2-year DFS/OAS rates of approximately 30% (Figure 2), justification enough to consider a curative IMRT approach also for this subgroup.

As in previous investigations on volumetric based outcome prediction [1] (Table VI in reference [1]) and [2], an inverse association between tGTV and disease control was also found in the here assessed patient segment with 'very large tumor volume': patients with tGTV > 130 cc tended towards inferior outcome, with nearly 40% achieving 3-year disease control (versus > 50% in patients with tGTV < 130 cc (NS), Table 3); again, this outcome data justify offering a curative combined modality treatment to compliant patients with very large tumor volumes.

Eight of 12 patients suspicious of initial M+ status were still alive at mean ~1 year (range 4-21 months) post treatment, enjoying beneficial treatment effects; the imaging diagnosis of limited M+ status should not be used as a criterion to exclude patients from loco-regional curative IMRT approach. Radiation is known as most effective local therapy for advanced non-resectable loco-regional disease, however requiring high doses, if possible enhanced by systemic therapy. In order to keep side effects as low as possible, IMRT should be used also in patients with radiologically suspicious distant lesions. Whether further investigation should be performed on suspicious M1 lesions has to be decided case by case.

The most unfavourable subgroup seems characterized by (elderly) patients with a tumour load of > 130 cc and unable to undergo any systemic treatment - this group however was too small to draw reliable conclusions from it (this question is under prospective evaluation at our institution).

### In sum

• patients with tGTV > 70 cc treated with IMRT and systemic therapy achieved 3-year DFS and OAS of approximately 50% and 60%, respectively

• (elderly) patients with tGTV > 70 cc treated with IMRT alone represented a subgroup which only marginally benefits from curative IMRT (3-year OAS/DFS ~25%/< 20%, 3-year DFS 17%); nevertheless, most patients experienced subjective benefit from radiation in terms of symptom relief, which is considered justifying IMRT at least aiming effective palliation

• (elderly) patients with very large tGTV > 130 cc treated with IMRT alone did worst (1-y OAS 0%), however, a larger sample size is needed for corroboration

## Conclusions

Tumour volume and eligibility to undergo systemic therapy were found important prognostic factors in patients with large tGTV load > 70 cc.

Larger sample sizes are required to test, if patients with tGTV > 130 cc and unable to undergo systemic therapy should be prevented from curative treatment approaches.

Radiobiological parameters may identify other criteria to define a palliative subgroup among the patients with very large tumour volumes.

## Conflicts of interest

The authors declare that they have no competing interests.

## Authors' contributions

GS conceived of the study and carried out its design, CG and GS draw the contours in all cases, approved all dose distribution plans and were responsible for the initial decision of a curative treatment approach in the individual patients. CG was involved in the data analysis and interpretation. TR decided on and supervised IC application in the assessed patients, and participated in the data acquisition. All authors read and approved the final manuscript.
